# Supplementary material for: Distinctive Microbial Signatures and Gut-Brain Crosstalk in Pediatric Patients with Coeliac Disease and Type 1 Diabetes Mellitus
Source: Int J Mol Sci. 2021 Feb 3;22(4):1511. doi: 10.3390/ijms22041511 (PMC7913584; doi:10.3390/ijms22041511)
Supplement: Supplementary file 1 [file ijms-22-01511-s001.zip › Suppl. Figures.pptx]

## Slide 1
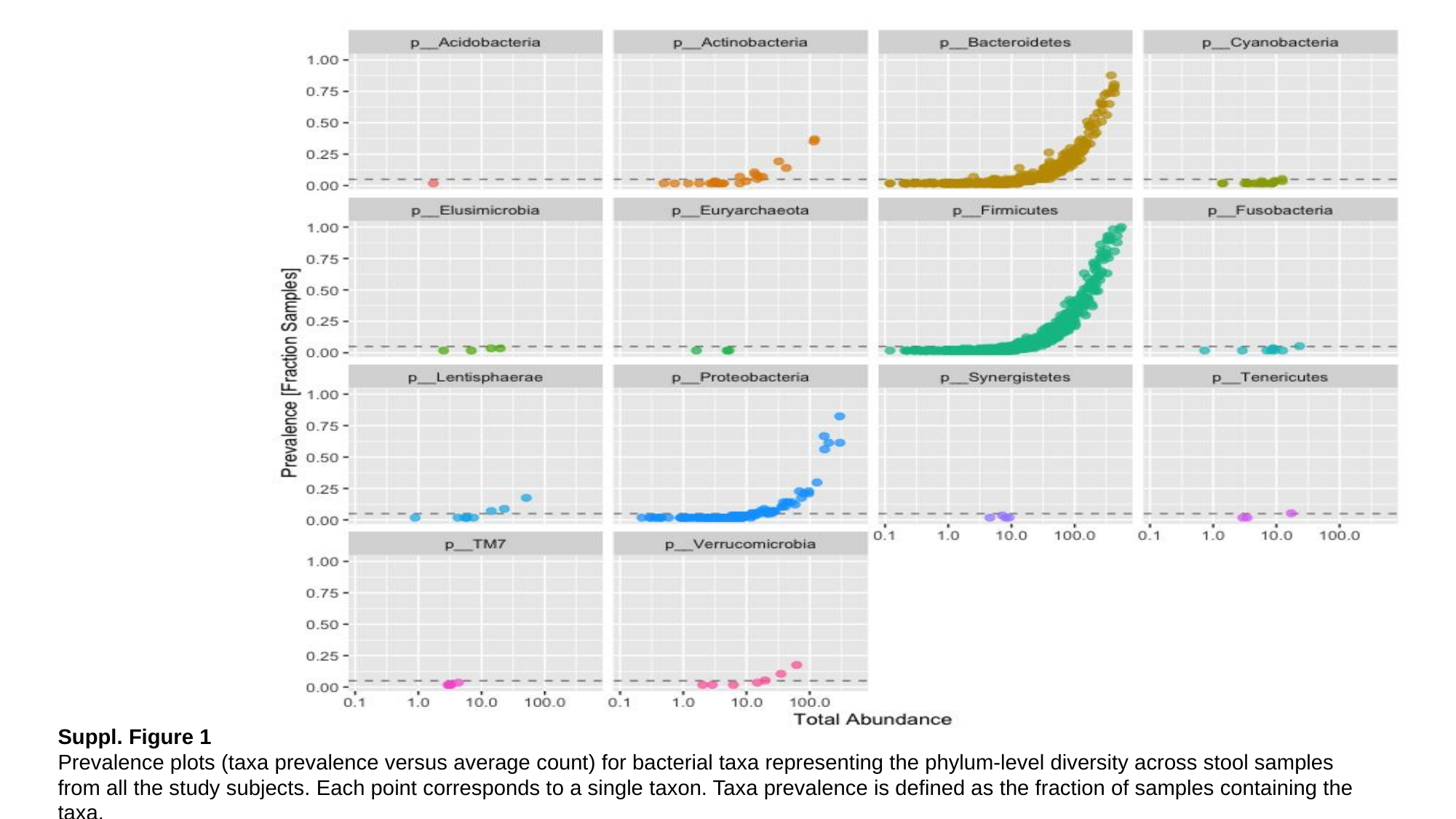

Suppl. Figure 1
Prevalence plots (taxa prevalence versus average count) for bacterial taxa representing the phylum-level diversity across stool samples from all the study subjects. Each point corresponds to a single taxon. Taxa prevalence is defined as the fraction of samples containing the taxa.

## Slide 2
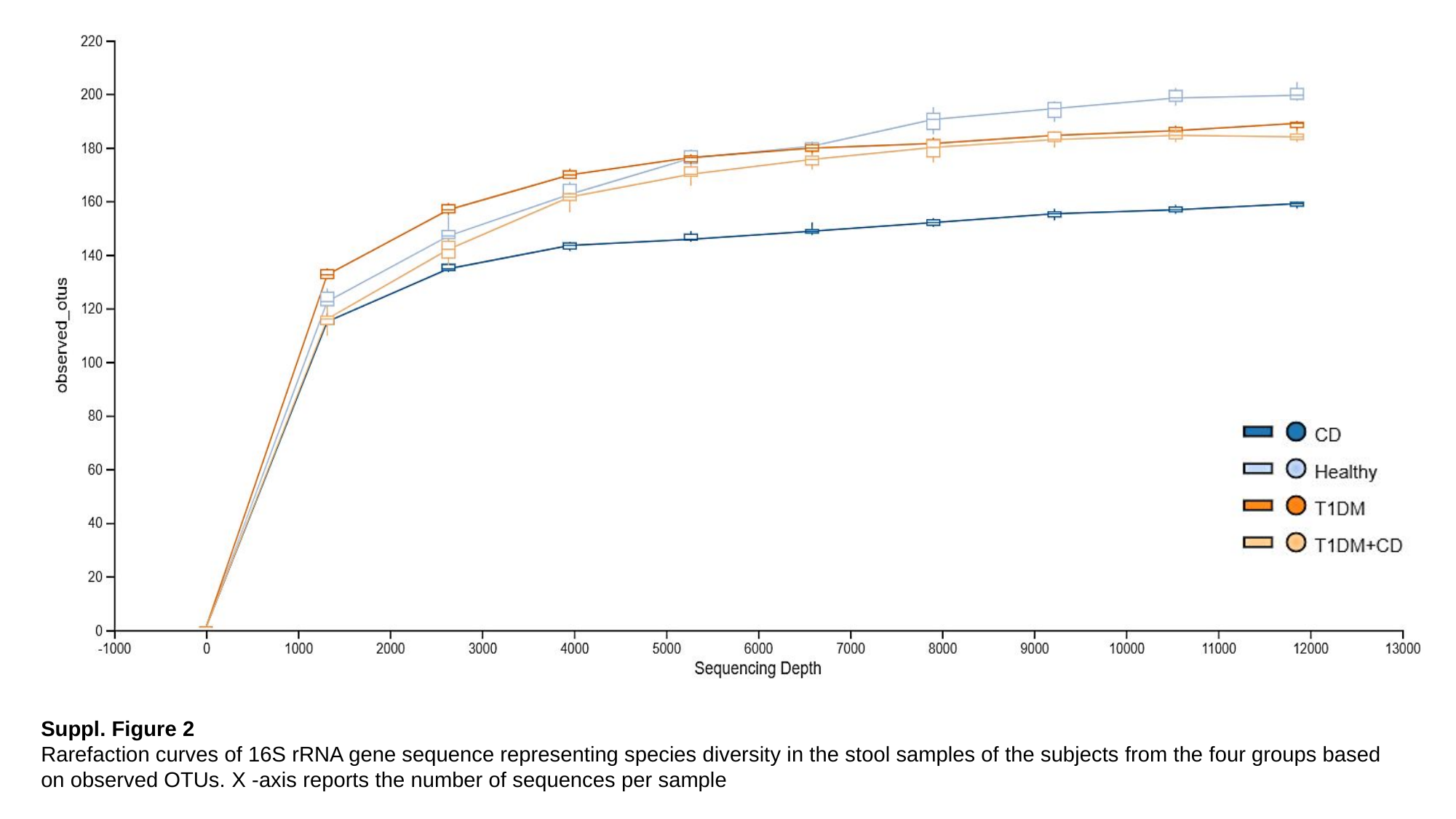

Suppl. Figure 2
Rarefaction curves of 16S rRNA gene sequence representing species diversity in the stool samples of the subjects from the four groups based on observed OTUs. X -axis reports the number of sequences per sample

## Slide 3
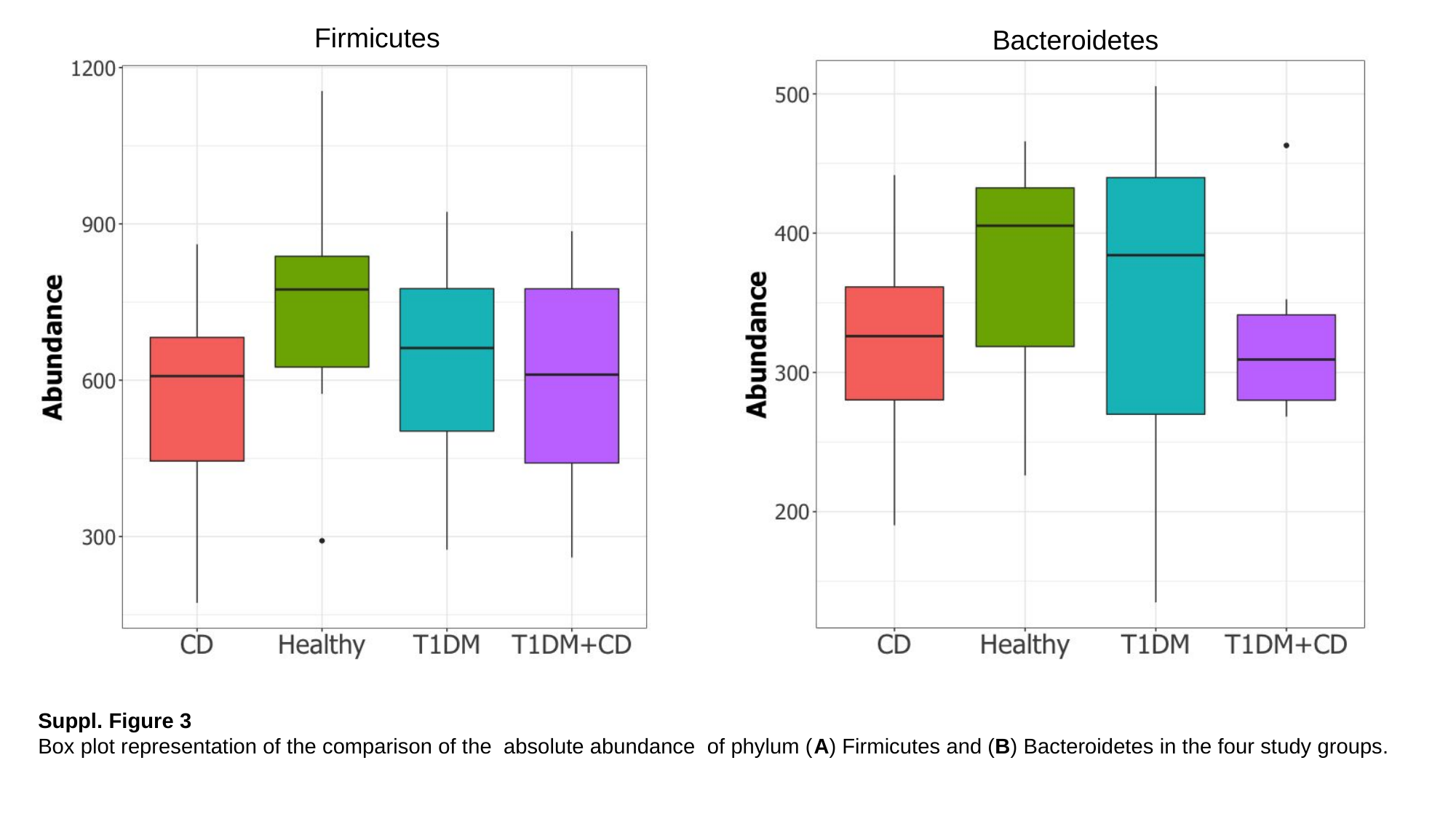

Firmicutes
Bacteroidetes
Suppl. Figure 3
Box plot representation of the comparison of the absolute abundance of phylum (A) Firmicutes and (B) Bacteroidetes in the four study groups.

## Slide 4
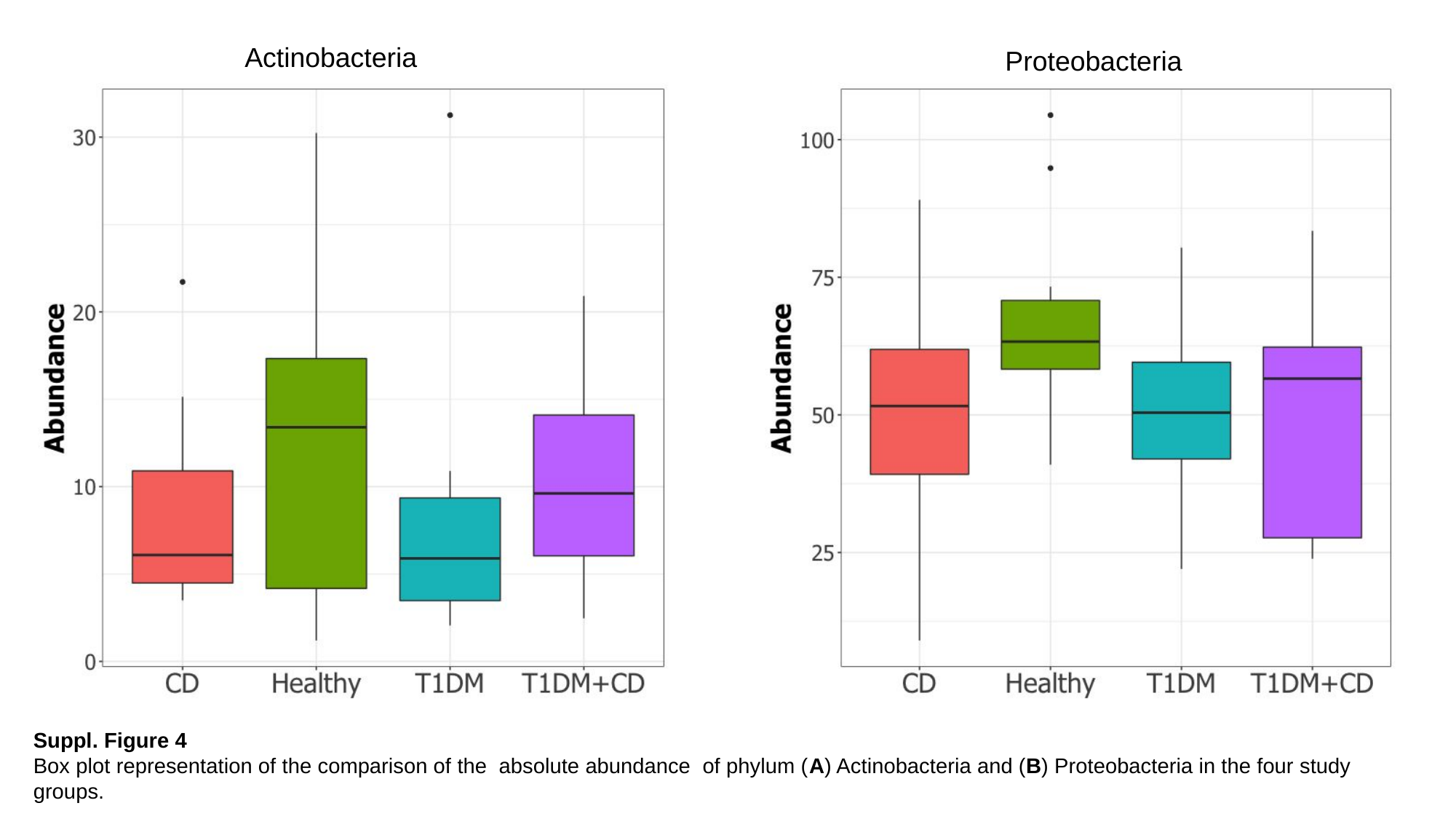

Actinobacteria
Proteobacteria
Suppl. Figure 4
Box plot representation of the comparison of the absolute abundance of phylum (A) Actinobacteria and (B) Proteobacteria in the four study groups.

## Slide 5
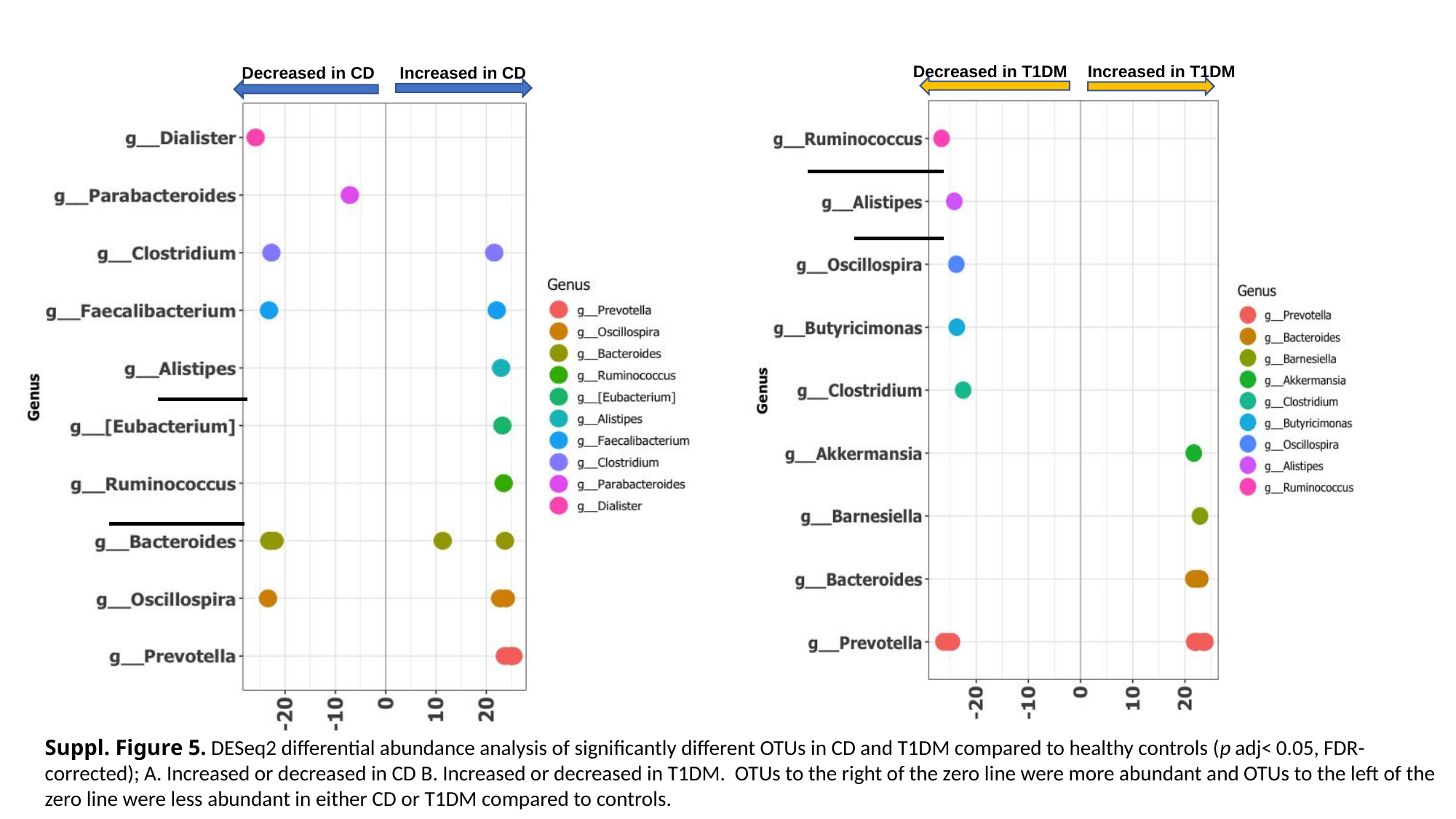

Increased in T1DM
Decreased in T1DM
Increased in CD
Decreased in CD
Suppl. Figure 5. DESeq2 differential abundance analysis of significantly different OTUs in CD and T1DM compared to healthy controls (p adj< 0.05, FDR-corrected); A. Increased or decreased in CD B. Increased or decreased in T1DM. OTUs to the right of the zero line were more abundant and OTUs to the left of the zero line were less abundant in either CD or T1DM compared to controls.

## Slide 6
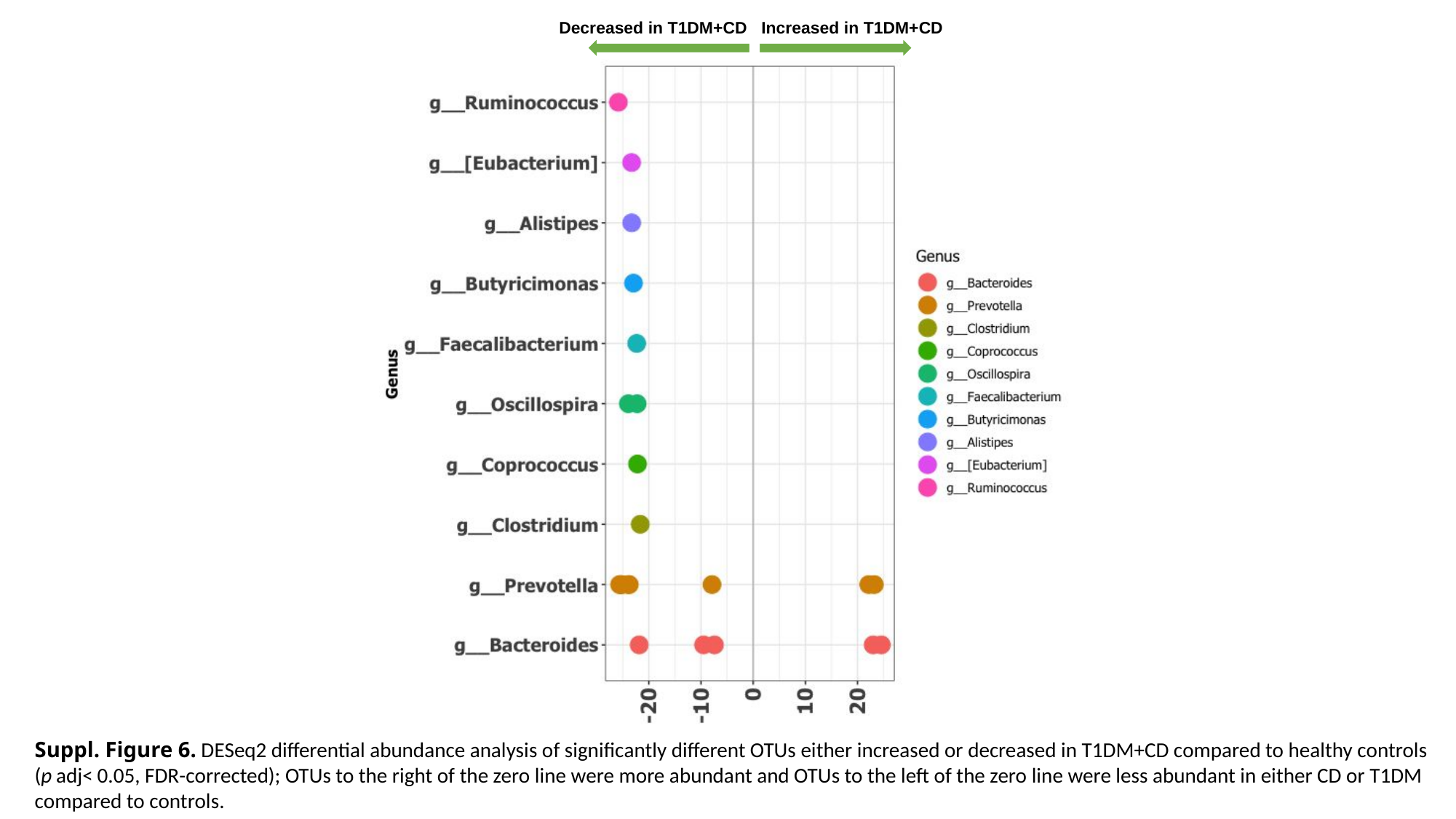

Decreased in T1DM+CD
Increased in T1DM+CD
Suppl. Figure 6. DESeq2 differential abundance analysis of significantly different OTUs either increased or decreased in T1DM+CD compared to healthy controls (p adj< 0.05, FDR-corrected); OTUs to the right of the zero line were more abundant and OTUs to the left of the zero line were less abundant in either CD or T1DM compared to controls.

## Slide 7
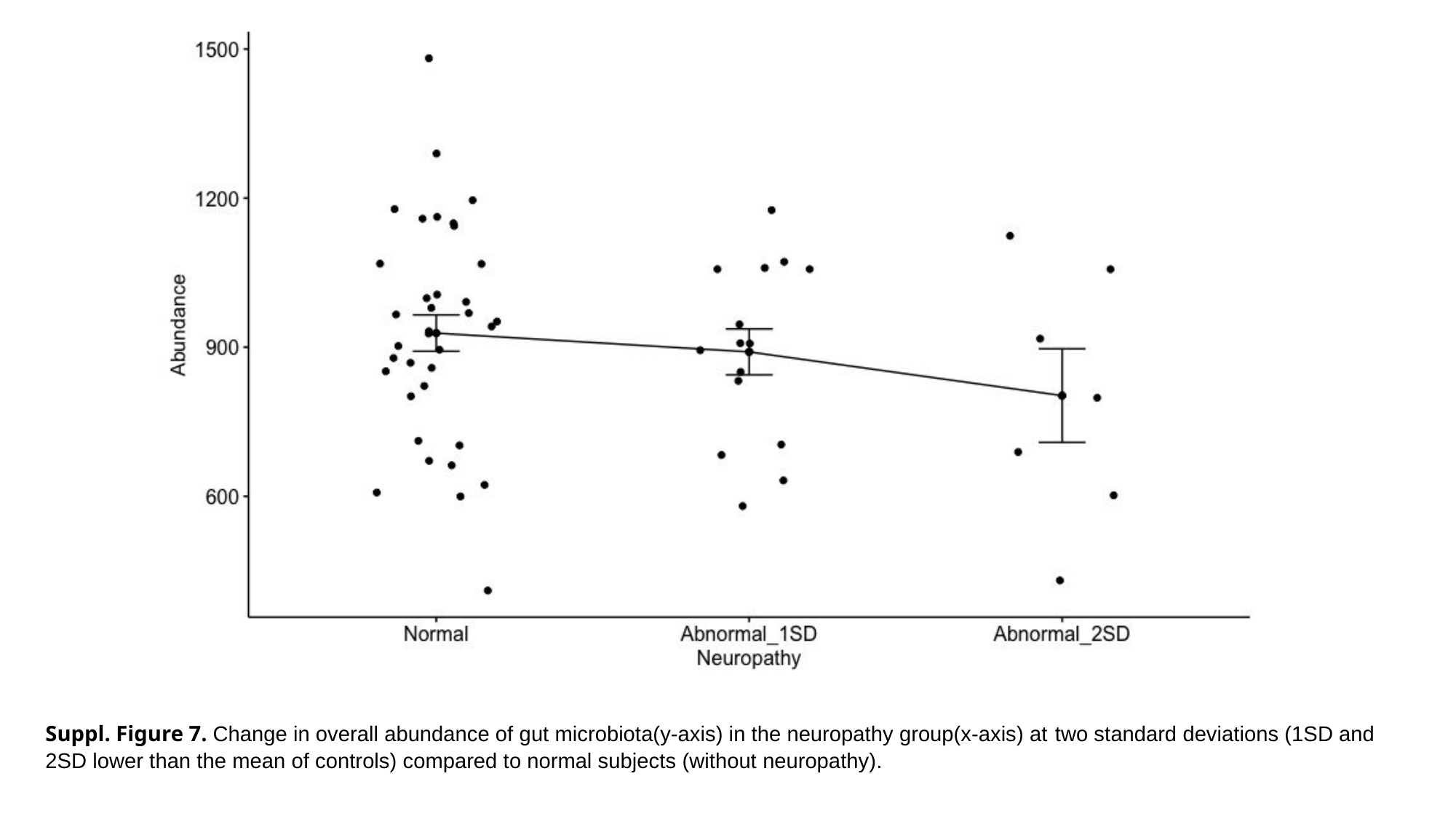

Suppl. Figure 7. Change in overall abundance of gut microbiota(y-axis) in the neuropathy group(x-axis) at two standard deviations (1SD and 2SD lower than the mean of controls) compared to normal subjects (without neuropathy).
